# Effect of Metformin on Doxorubicin-Induced Memory Dysfunction

**DOI:** 10.3390/brainsci10030152

**Published:** 2020-03-07

**Authors:** Ibrahim Alharbi, Hindi Alharbi, Yasser Almogbel, Abdullah Alalwan, Ahmad Alhowail

**Affiliations:** 1 Department of Pharmacology and Toxicology, College of Pharmacy, Qassim University, Buraydah 51452, Saudi Arabia; pharmacy33@hotmail.com (I.A.); hindialharbi@gmail.com (H.A.); 2 Department of Pharmacy Practice, College of Pharmacy, Qassim University, Buraydah 51452, Saudi Arabia; y.almogbel@qu.edu.sa (Y.A.); alalwan@qu.edu.sa (A.A.)

**Keywords:** doxorubicin, memory impairment, chemobrain, metformin

## Abstract

Doxorubicin (DOX) is widely used to treat many types of cancer; however, it is associated with chemotherapy-related complications such as cognitive dysfunction, known as chemobrain. Chemobrain affects up to 75% of cancer survivors, and there are currently no available therapeutic options. This study aims to examine whether metformin (MET) can protect against the neurotoxicity caused by DOX treatment. Forty male rats were divided into four groups (10 rats/group): control, DOX, DOX + MET, and MET. Rats treated with DOX received five doses of 4 mg/kg DOX weekly (cumulative dose: 20 mg/kg). For the DOX-MET and MET groups, MET (3 mg/mL) was dissolved in drinking water. Behavioral and glucose tests were performed one day after treatment was completed. We found DOX (4 mg/kg/week, 5 weeks) caused learning and memory impairment in the Y-maze, novel object recognition, and elevated plus maze behavioral tests. MET did not rescue these DOX-induced memory impairments. Neither DOX nor MET nor MET + DOX altered glucose levels following the treatment. In summary, DOX treatment is associated with memory impairment in rats, but MET does not rescue this cognitive dysfunction.

## 1. Introduction

Recent advancements in chemotherapy have shown success in eradicating various types of cancer. The main mechanism underlying the action of chemotherapeutic agents is cytotoxicity. However, the toxicity associated with chemotherapy leads to several acute and chronic adverse side effects [1,2,3,4,5], including cognitive dysfunction, which is referred to as chemobrain [6]. The cognitive dysfunction can vary from moderate to severe and can affect patients’ emotional, behavioral, and mental status, which consequently influences their ability to concentrate, multitask, and memorize [7]. Currently, there are over 16 million cancer survivors in the USA, and this number is expected to increase to 22 million by 2030 [8]. As cognitive impairments affect up to 75% of cancer survivors [6], chemobrain remains a major clinical challenge. Unfortunately, therapeutic strategies to address neurotoxicity during and after chemotherapy are limited.

Preclinical and clinical studies have revealed chemotherapy is associated with impairment of cognitive function. For example, chronic use of cyclophosphamide (CYP), doxorubicin (DOX), and cisplatin were shown to severely impair hippocampus-dependent cognitive function in rodents [9]. These cognitive deficits were linked to neurogenesis, alterations in protein function, and inflammation. Indeed, we previously showed that acute DOX exposure (which is commonly used to treat various types of cancer [10]) is associated with reduced hippocampal long-term potentiation, increased lipid peroxidation, and apoptosis [11]. Moreover, despite the low ability of DOX to cross the blood–brain barrier, acute DOX treatment has been shown to negatively affect hippocampal cell proliferation [12]. Furthermore, the combination of DOX and CYP has been reported to impair cognitive function by increasing the phosphorylation of AKT and extracellular signal-regulated kinase 1/2 (Erk1/2) proteins, as well as promoting inflammation [13]. Chemotherapy agents such as DOX can also generate reactive oxygen species (ROS), which, in turn, may induce oxidative stress [14]. Finally, using animal models of chemobrain and in vivo models of cognitive impairment, we previously showed that insulin signaling could be one of the factors mediating the cognitive dysfunction in chemobrain [15]. Together these findings indicate that exposure of the brain and central nervous system to chemotherapy can alter brain function, although the precise mechanism requires further investigation.

Growing evidence has documented the beneficial effects of metformin (MET), a commonly used anti-diabetic drug, on multiple diseases other than diabetes. For instance, the long-term use of MET has been associated with anticancer effects [16] and extending lifespan [17,18]. However, research on the effects of MET on cognitive function has to date produced inconclusive findings. Indeed, some studies have reported beneficial effects of MET on memory function in rodents [1,9], while others demonstrated the opposite effect when the drug was given to healthy mice [15]. MET has been shown to decrease the risk of Alzheimer’s disease [19], and yet, potential adverse effects on cognitive performance have been reported in diabetic patients when MET is used chronically [19,20]. Despite this controversy, several lines of evidence suggest MET may be useful in the prevention of chemobrain, likely by suppressing inflammatory activity and reducing oxidative stress [21]. Indeed, MET co-administration was shown to ameliorate chemotherapy-induced nephrotoxicity and hepatotoxicity, as well as to improve memory impairment following cisplatin and CYP treatments [9]. Furthermore, using an animal model of CYP-induced chemobrain, we previously showed behavioral impairments were rescued when MET was co-administered with CYP [15]. Thus, we hypothesize that MET may prevent DOX-induced chemobrain in a similar manner to CYP-induced chemobrain. In particular, as DOX was previously reported to alter insulin receptor signaling [22], we propose MET may improve memory dysfunction in DOX-treated rats by enhancing insulin sensitivity and signaling [23,24].

Therefore, this study aims to investigate whether MET co-administration can protect against DOX-induced cognitive impairment using rat models.

## 2. Materials and Methods

### 2.1. Animals

Forty male rats (200–250 g) were individually housed and maintained on a 12-h light/dark cycle with free access to food and water. Animals were observed daily, and their body weights measured every 3 days. All behavioral tests were performed during the light phase of the cycle. The ethics and protocol of this research were approved by the Research Unit at the College of Pharmacy at Qassim University. This is no informed consent required for this study.

### 2.2. Drug Administration

Animals were divided into four groups: DOX, MET, DOX + MET, and control groups. Rats received an intraperitoneal (i.p.) injection of 4 mg/kg DOX weekly for 5 weeks (total dose: 20 mg/kg). MET was dissolved in the rats’ drinking water at a concentration of 3 mg/mL. MET was administered daily, starting the day before DOX treatment. The rats underwent behavioral tests after receiving five scheduled DOX doses.

### 2.3. Y-Maze Test

The Y-maze test measures an animal’s ability to recognize places they have already explored and their ability to explore new places [14]. We used the Y-maze test to assess the animals’ ability to perform hippocampus-dependent tasks and their working memory. The Y-maze was made of wood (dimensions 50 × 10 × 18 cm), with three arms placed at 120°. The arms were painted brown to ensure easy visualization. The apparatus was placed on the floor. Light was provided from above to ensure equal light distribution. In the training session, the animals were allowed to freely explore two arms for 15 min. During the second session (5-min duration), the animals were allowed to explore the entire maze, including the novel arm. The time between the two sessions was 3 h. The test sessions were video-recorded to determine the time spent by the animals in each arm and the number of entries (note: an animal was considered to have entered an arm if half of its body entered).

### 2.4. Novel Object Recognition Test

We used the novel object recognition (NOR) test to evaluate memory function [25]. The test apparatus is a wooden box (dimensions 40 × 40 × 40 cm) with an open top. The familiarization objects were two white teacups, and the novel object was a black box (of size equal to the teacup). In the training session, the rats were allowed to explore the two teacups for 10 min and then returned to their cages. In the second session (3 h later; 5-min duration), one of the teacups was replaced with the novel object and the time spent exploring the novel object was recorded using a video camera and a stopwatch [26].

### 2.5. The Elevated Plus Maze Test

The elevated plus maze (EPM) test is used to measure anxiety as well as learning and memory processes. The wooden apparatus consists of two opposing arms: the open arms (50 × 10 cm) and the closed arms (50 × 10 cm). The height of the sidewalls of the closed arms was 30 cm. The central platform between all arms measured 10 cm^2^. The maze was placed 50 cm above the floor. In the training session, the rat was placed at the end of an open arm, facing the central platform, and allowed to explore the apparatus for 10 min. Three hours later, the rat was placed in the same spot as in the training session, and the transfer latency time (i.e., the time it took the rat to move from the open arm into either of the closed arms) and total time spent in the closed arms were recorded using a video camera [27].

### 2.6. Blood Glucose Test

The blood glucose test was used to evaluate the glucose levels of rats. The tail vein was injured with a clean, sterile needle to obtain optimum-quality blood. An Accu-Chek glucometer with strips was used to test the blood glucose level based on the manufacturer’s instructions.

### 2.7. Statistical Analysis

All results are presented as means ± standard error of the mean (SEM) and were analyzed using Graphpad Prism 5 software. The survival rate, Y-maze, NOR, EPM, and blood glucose data for each group were analyzed using one-way analysis of variance followed by Dunnett’s analysis. All the treatment group data were compared with that for the control group. A *p*-value <0.05 was considered statistically significant.

## 3. Results

### 3.1. Effect of DOX and MET on Mortality and Body Weight

**Figure 1:**
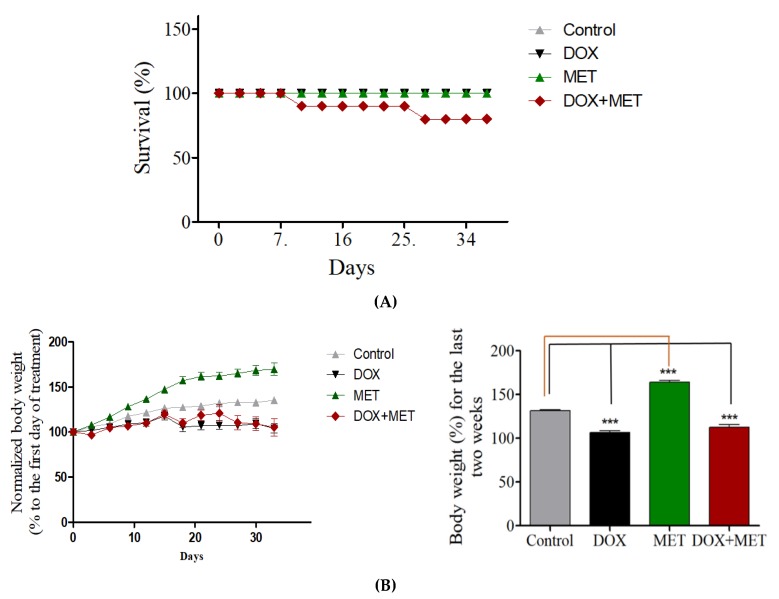
(**A**) Effects of doxorubicin (DOX) and metformin (MET) on survival rate of rats. (**B**) Effects of DOX and MET on rat body weight.

Chronic DOX treatment did not affect the survival rate of rats; however, we found a higher incidence of death among the rats that received both DOX and MET (Figure 1A). In total, 10% of the rats treated with DOX and MET died after the first week of treatment. This percentage increased to 20% in the fourth week of DOX and MET treatment. The study was terminated after the fifth week of treatment. The bodyweight of DOX-treated rats and DOX + MET-treated rats was significantly reduced compared with control rats (Figure 1B).

### 3.2. Effect of DOX and MET on Y-Maze Performance

**Figure 2:**
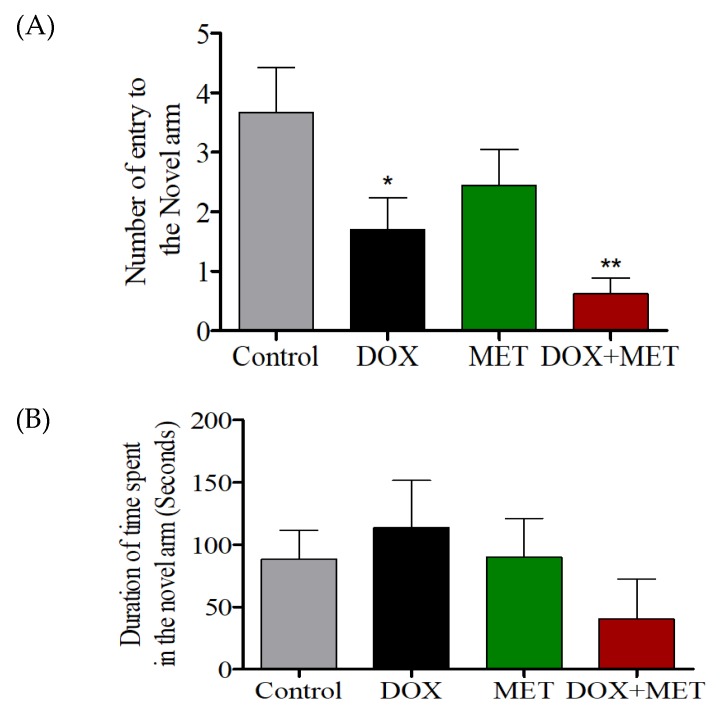
(**A**) Effects of DOX and MET on the number of entries into the novel arm in the Y-maze test. (**B**) Effects of DOX and MET on the total time spent in the novel arm in the Y-maze test.

DOX-treated rats showed significantly fewer entries into the novel arm than DOX + MET-treated rats (Figure 2A). However, when MET was used alone, only a slight reduction in the number of entries was observed, which was not statistically significant (Figure 2A). There was no significant difference in the time spent in the novel arm among the four groups, indicating all rats could not distinguish the novel arm from the other arms (Figure 2B).

### 3.3. Effects of DOX and MET on NOR Test Performance

**Figure 3:**
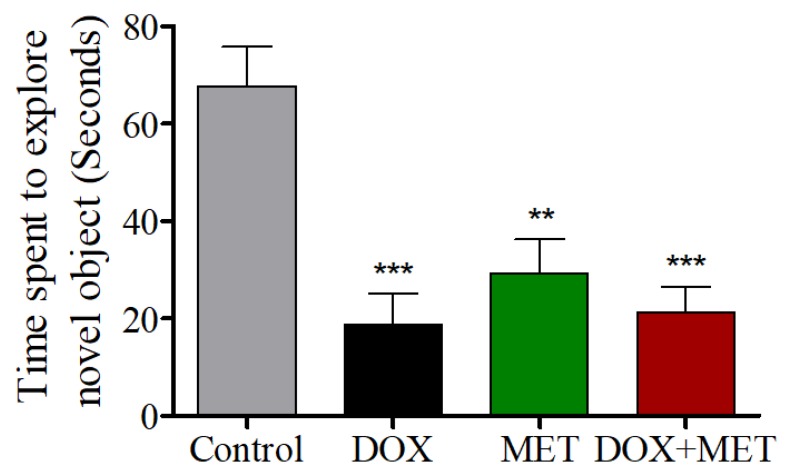
Effects of DOX and MET on novel object recognition.

The DOX, DOX + MET, and MET-alone groups were significantly different from the control group in the NOR test, suggesting DOX and/or MET potentially alter memory function in rats (Figure 3).

### 3.4. Effects of DOX and MET on EPM Test Performance

**Figure 4:**
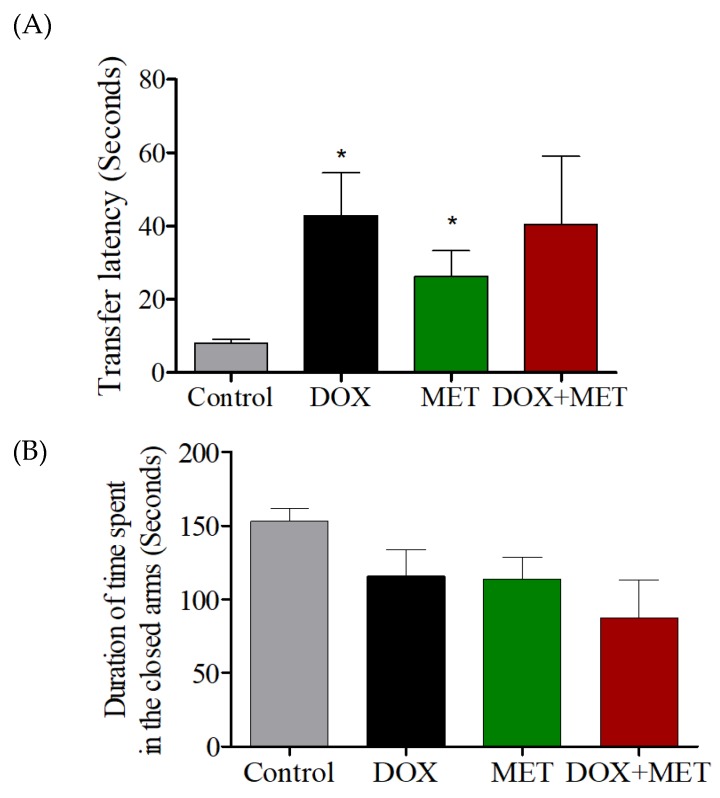
(**A**) Effects of DOX and MET on the transfer latency time in the elevated plus maze test. (**B**) Effects of DOX and MET on the total time spent in the closed arms in the elevated plus maze test.

The transfer latencies in the DOX-treated and MET-treated groups were significantly higher than those in the control group (Figure 4A). Similarly, the transfer latency in the DOX + MET treated group was higher than in the control group, albeit not significantly. This suggests that memory was impaired in the DOX, MET-, and DOX + MET-treated groups (Figure 4A). Meanwhile, there was no significant difference in the total time spent in the closed arms in the EPM test among the four groups (Figure 4B).

### 3.5. Effects of DOX and MET on Blood Glucose Levels

**Figure 5:**
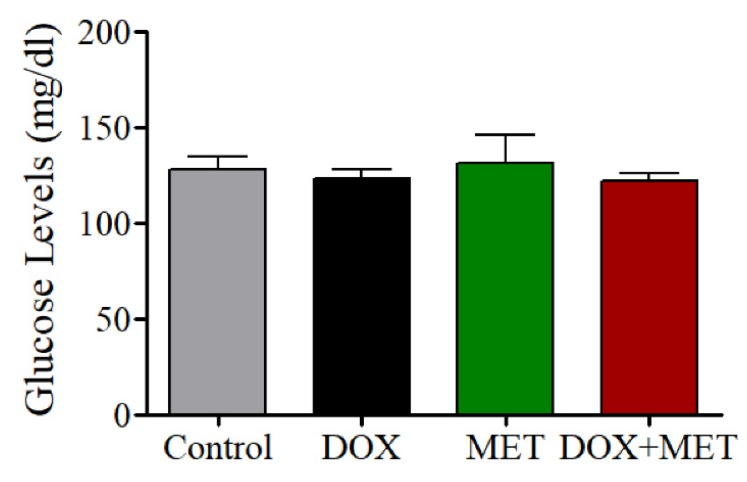
Effects of DOX and MET on rat blood glucose levels.

Glucose levels were assessed one day after treatment was concluded. As shown in Figure 5, DOX- and MET-treated rats did not show a significant change in their glucose levels compared with controls, indicating that DOX does not affect glucose levels during short-term treatment. There was a slight decrease in the glucose levels in DOX-treated rats, which may have been caused by a reduction in food consumption as a result of the DOX treatment.

## 4. Discussion

Preclinical and clinical studies have shown that DOX can alter cognitive functions [23,24]. Here we found that weekly treatment with DOX for 5 weeks resulted in impairment of spatial memory in rats. In addition, a previous study using rats showed DOX impairs memory function in novel place recognition [6], and we also detected a specific impairment in the Y-maze task after DOX treatment in rats. We also hypothesized that MET may have a protective effect on DOX-induced impairments in memory function by blocking the metabolic stress response [28], inhibiting the PI-3-kinase–Akt–mTOR (PI3K/Akt/mTOR) pathway, and/or reducing ROS-mediated stress [29]. However, unlike our previous findings using a rat model of CYP-induced cognitive impairment [15], MET did not ameliorate the DOX-induced memory impairment in this study.

Although we hypothesized that MET could improve memory dysfunction in Alzheimer’s disease patients by enhancing insulin sensitivity and signaling [26], we observed that chronic treatment with MET alone actually induced cognitive deficits in rodents [15]. In addition, in the current study, MET failed to rescue cognitive dysfunction following DOX treatment in rats. We also found no significant difference in glucose levels among the four treatment groups, which suggests DOX may not affect the expression of insulin receptors. As DOX does not appear to affect insulin sensitivity, MET likely has little effect on improving glucose-mediated memory impairment, which is supported by our findings.

During NOR tasks, memory was impaired in DOX-treated animals, as well as in the MET- and DOX + MET-treated groups, confirming that cognitive functions were affected. Rats spent less time exploring the novel object in the DOX, MET, and DOX + MET groups, indicating memory was impaired as a result of the treatments. In addition, in the EPM, all treatment groups had longer transfer latency times compared with the control group. However, the total time spent in the closed arms was similar to that in controls, indicating the longer transfer latency time was due to memory issues and not a result of lethargy following treatment. Together, these data suggest that DOX treatment can impair memory function that relies on the hippocampus, and MET treatment failed to alleviate these deficits.

We also found a higher incidence of death among rats receiving both DOX and MET [30]. Therefore, co-administration of MET with DOX may increase drug toxicity or produce a synergistic effect against all cells leading to death. This is in contrast to our previous finding, in which the survival rate of DOX-treated mice improved when MET was used [18]. However, in our previous study, only a single dose of DOX and MET was used (25 mg/kg) compared with the chronic DOX treatment (five doses at 4 mg/kg) used in this study. Therefore, the treatment regimen may explain the differences in the observed results. Moreover, our previous study was performed in mice, not rats.

## 5. Conclusions

In conclusion, our study supports the results of previous studies that show memory loss due to DOX treatment using behavioral tests. Although MET can reduce the cytotoxic effects of several chemotherapy agents (such as nephrotoxicity, hepatotoxicity, and cardiotoxicity [9,31,32]), it failed to reduce neurotoxicity induced by DOX treatment in this study. Therefore, further clinical studies are needed to investigate the protective effects of MET on neurotoxicity in humans.
